# Evaluation of chemotherapy-induced nausea and vomiting in low, moderate, and highly emetogenic schemes between sexes

**DOI:** 10.1007/s00520-025-09319-7

**Published:** 2025-03-10

**Authors:** Marta Albanell-Fernández, Ma Carmen Rodríguez Mues, Carolina Figueras, Mariana Altamirano, Inés Monge-Escartín, Gisela Riu-Viladoms, Esther Carcelero San Martín, Mª Lourdes Corominas Bosch, Lydia Gaba García

**Affiliations:** 1 Pharmacy Department, Division of Medicines, Hospital Clinic of Barcelona, Barcelona, Spain; 2 Department of Medical Oncology, Hospital Clinic of Barcelona, Barcelona, Spain; 3 Department of Physiological Science, School of Medicine, Universitat de Barcelona (UB), L’Hospitalet de Llobregat, Barcelona, Spain

**Keywords:** Chemotherapy-induced nausea and vomiting (CINV), Supportive care, Male, Female, Antiemetics, Cancer

## Abstract

**Purpose:**

Sex influences chemotherapy-induced nausea and vomiting (CINV). However, in clinical practice, males and females receive the same antiemetic prophylaxis. We compared CINV between sexes in patients with different emetic risk schemes and evaluated the predisposing factors and main adverse effects caused by antiemetics.

**Methods:**

Prospective observational study conducted in a tertiary-care hospital from February 2023 to May 2024 in patients starting chemotherapy or a new treatment line. CINV was evaluated using MASCC antiemetic tool, in acute (< 24 h) and delayed phases (24–120 h). Results were analyzed using *χ*^2^ test or Fisher’s exact test. The primary endpoint was complete response (CR) rate, defined as no CINV and no use of rescue medication. Univariate and multivariate logistic regressions were used to identify patient-related risk factors associated with non-CR.

**Results:**

A total of 176 completed questionnaires (CQ): 94 for males and 82 for females were collected. The proportion of males who remained emesis-free was superior to females in the acute phase (100% versus 92.7%, *p* = 0.009). Likewise, a higher proportion of males remained nausea-free in the acute (91.5% versus 79.3%, *p* = 0.021) and delayed phase (90.4% versus 79.3%, *p* = 0.037). In females, young age (< 60 years) and previous nausea and vomiting during pregnancy may contribute to non-CR. A high proportion of patients reported adverse events like constipation and insomnia. Females suffered more constipation than males (52.4% versus 37.2%, *p* = 0.043).

**Conclusion:**

Females experienced more CINV than males, with the consequences that entail. Antiemetic prophylaxis should be personalized, considering sex and age and not only the chemotherapy emetic potential.

## Introduction

Chemotherapy-induced nausea and vomiting (CINV) is one of the adverse events that most interferes with the quality of life of oncology patients [1]. Poorly controlled CINV negatively impacts patients’ quality of life and compromises treatment adherence. Patients experiencing CINV may be less likely to complete their prescribed chemotherapy regimen [1–3]. Inadequately controlled CINV can lead to dose reductions, delays in chemotherapy, and adjustments in antiemetic regimens [4, 5], which leads to a worse outcome in cancer treatment [6].

A recent study reported an incidence of 57% of CINV in patients with highly/moderately emetogenic chemotherapy [7], which agrees with previous data reporting 60% for nausea and 36% for emesis [3]. Acute onset-CINV occurs within 24 h of initial chemotherapy, while delayed-onset occurs > 24 h and may last up to 5 days [7, 8]. Delayed CINV is typically more prevalent and more difficult to control, as it is less responsive to antiemetic therapy [8–12].

The prevention of CINV is an important element of supportive care in cancer [7, 12]. The most important factor influencing the severity of CINV has been reported to be the emetic potential of chemotherapy [13, 14]. To address it, a four-tiered antiemetic prophylaxis is employed based on the emetic risk associated with specific chemotherapy regimens [8, 15]. However, other predisposing factors related to the patient have been described, like young age, female sex, poor functional status, cancer type, pregnancy-related nausea and vomiting, susceptibility to motion sickness, and non-habitual alcohol and tobacco consumption [6, 16–19].

In a society where there is an increasing integration of gender perspectives, to date, there have been no prospective studies that have assessed the incidence of CINV based on sex. In daily clinical practice, males and females are treated equally for CINV prevention. Even though females, due to their baseline condition and a higher predisposition to present risk factors, have a higher susceptibility to suffering CINV [6, 20–22]. We aimed to prospectively compare the incidence of CINV between sexes in patients initiating chemotherapy or a new treatment line with different emetic risk schemes. As secondary objectives, we examined the main predisposing factors that influence CINV in each sex and analyzed the incidence of the main adverse effects caused by the antiemetic drugs.

## Methods and materials

### Study design and setting

This prospective observational study was conducted in a tertiary care hospital from February 2023 to May 2024. We included outpatients with solid tumors who received chemotherapy in the Oncology Therapy Unit. There was no pre-specified limit of consecutive chemotherapy cycles for each patient. The research was approved by the Institutional Ethics Committee of Hospital Clinic of Barcelona (Reg: HCB/2023/0014). The study was conducted in compliance with the Declaration of Helsinki. All patients provided written informed consent before treatment initiation.

### Patient sample

Enrollment eligibility criteria included ≥ 18 years with a diagnosis of gastric, pancreatic, lung, and colon cancer, who initiated a new chemotherapy scheme. We selected only tumors with a similar incidence between men and women to make the groups comparable [23]. Both naïve chemotherapy patients and those initiating a new line of treatment after the previous progression were included. Additional eligibility criteria comprised an Eastern Cooperative Oncology Group performance status (ECOG) of 0 to 3. Patients were excluded if they were already under treatment at the time the study was initiated, if they had known allergies or intolerances to any of the drugs used in antiemetic prophylaxis, if they were already enrolled in a clinical trial, and if the antiemetic treatment had been modified before the start of the first cycle of chemotherapy or during the treatment.

### Data collection and study measures

The following independent variables were recorded from the medical history and the survey: demographic data, susceptibility to CINV (pregnancy-related nausea and vomiting, motion sickness, and alcohol and/or tobacco consumption), tumor type, setting of treatment (neoadjuvant/adjuvant or metastatic), chemotherapy treatment line, cycle number, emetic potential of the regimen, and ECOG.

The MASCC Antiemesis Tool (MAT) was used to measure CINV objectively [21]. The MAT questionnaire records nausea and emesis separately, differentiating in the acute phase (first 24 h) and in the delayed phase (24–120 h after chemotherapy). It asks whether the patient has suffered emesis and/or nausea, as well as the number of episodes.

On the visit before starting the chemotherapy scheme, the advanced practice nurse (APN) assigned to the patient was in charge of explaining the study and giving the necessary instructions for completing the questionnaires. The APN completed the predisposing factors survey and gave the patient the informed consent, which should be returned along with during that visit. She also provided the MAT questionnaire and a questionnaire about the possible antiemetic adverse events to be completed in subsequent cycles of chemotherapy. The patient filled out both questionnaires after each cycle and returned them the following cycle. Then, they were given a new questionnaire for the new cycle.

The primary endpoint was the achievement of complete response (CR) to antiemetic prophylaxis, which was defined as no CINV (nausea and emesis) events and no use of rescue medication over 120 h after chemotherapy. Treatment failure was defined as the presence of emesis or nausea in any of the phases (acute or delayed) or any administration of rescue medication. The assessment of efficacy was based on patients’ answers to the questionnaires.

### Statistical analysis

According to the meta-analysis conducted by Mosa et al., the summary odds ratio was estimated to be 2.79 (95% CI 2.26–3.44) [24], assuming that 15% of males (control group) will experience non-CR. Using a risk of 5%, a power of 80%, and a similar proportion of men and women (1:1), the estimated sample size was 176 completed questionnaires (88 for each group: males and females).

Descriptive statistics were used to summarize patient demographics and survey responses. Differences in the proportion of patients that reported acute and delayed CINV between sexes were analyzed using the *χ*^2^ test or Fisher’s exact test, when corresponding. The proportion of emesis and nausea in each of the phases was analyzed separately. A *p* < 0.05 was considered statistically significant.

Univariate and multivariate logistic regressions to identify patient-related risk factors associated with a non-CR in the overall phase were performed. All predisposing factors to CINV development were included as independent variables. The variables included were age (recoded as young if < 60 years and older if ≥ 60 years), emetic potential risk (low, moderate, and high), line of treatment (first line or other lines), ECOG (0, 1, 2), pregnancy-related nausea and vomiting (yes or no), susceptibility to motion sickness (yes or no), history of habitual alcohol intake (yes or no), and history of smoking (yes or no). The statistical criteria for accepting variables in the model were *p* < 0.05. Odds ratios and 95% confidence intervals (CI) for non-CR were calculated.

The proportions of adverse effects (insomnia, headache, and constipation) in each group were compared with the *χ*^2^ test or Fisher’s exact test, as appropriate. A *p* < 0.05 was considered statistically significant.

All statistical analyses were performed with STATA/BE 17.0.

## Results

A total of 90 patients were screened to participate in the study, although only 54 returned completed the MASCC questionnaire and were therefore included in the analysis, which meant 176 completed questionnaires (CQ): 94 CQ for males and 82 CQ for females. The mean age (SD) was 67.2 (8.5) years for males and 66.0 (5.8) years for females. Patients received between 1 and 12 cycles, according to the chemotherapy scheme. In the male group, the most frequent diagnosis was lung cancer (58.5%) while in the female group, was pancreatic cancer (48.8%). Palliative setting was most frequent in both groups (73.4% in males and 56.1% in females), and most patients were chemotherapy naïve (95.7% males and 80.4% females). The performance status (ECOG score) was quite similar between groups. As predisposing/protective factors for CINV development, 24.4% of females had a history of pregnancy-related nausea and vomiting, while in males, habitual alcohol intake (27.7%) and habitual smoking (22.3%) were the more frequent factors. In both groups, a high proportion of patients (56.4% in males and 43.9% in females) had high emetic chemotherapy. Patient descriptive statistics are summarized in Table 1.

**Table 1:** Demographic and clinical characteristics of patients

Characteristics	Groups	
	Male (*n* = 29)	Female (*n* = 25)
MASCC completed questionnaires—*n* (%)	94	82
Age in years (mean ± SD)	67.2 ± 8.5	66.0 ± 5.8
Cancer type—*n* CQ (%)		
Lung	55 (58.5)	25 (30.5)
Colorectal	7 (7.5)	3 (3.7)
Gastric	13 (13.8)	3 (3.7)
Pancreas	19 (20.2)	40 (48.8)
Other	0 (0)	11 (13.4)
Setting—*n* CQ (%)		
Neoadjuvant/adjuvant	25 (26.6)	36 (43.9)
Metastatic	69 (73.4)	46 (56.1)
Treatment line:		
First line	66 (95.7)	37 (80.4)
Second line	3 (4.3)	9 (19.6)
ECOG—*n* CQ (%)		
0	32 (34.0)	27 (32.9)
1	58 (61.7)	44 (53.7)
2	4 (4.3)	10 (12.2)
3	0 (0)	1 (1.2)
Predisposing factors—*n* CQ (%)		
History of nausea and vomiting during pregnancy	-	19 (24.4)
Motion sickness	0 (0)	3 (3.7)
Habitual alcohol intake	26 (27.7)	4 (4.9)
Habitual smoking	21 (22.3)	10 (12.2)
Chemotherapy scheme emetic risk—*n* CQ (%)		
Low	19 (20.2)	29 (35.4)
Moderate	22 (23.4)	17 (20.7)
High	53 (56.4)	36 (43.9)

*ECOG* Eastern Cooperative Oncology Group, *CQ* completed questionnaires, *SD* standard deviation

**Fig. 1:**
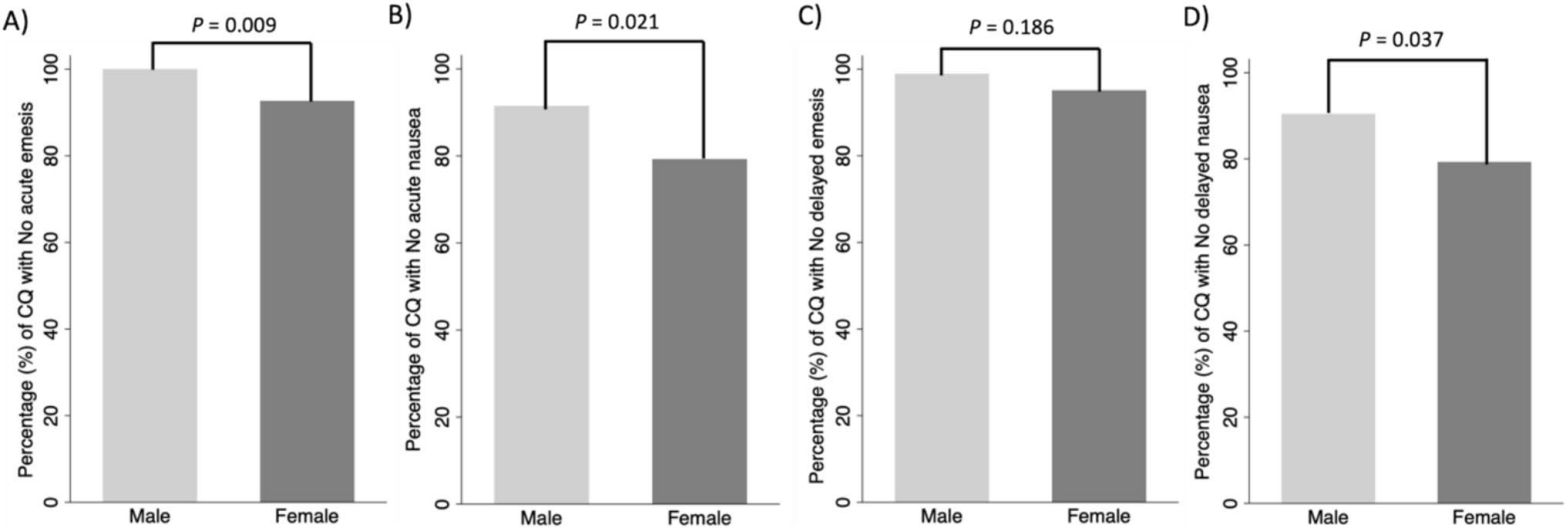
Graph of CINV results according to sex in the acute and late phases of completed questionnaires. **A** Percentage of no acute vomiting during the first 24 h after chemotherapy for each sex. **B** Percentage of no acute nausea during the first 24 h after chemotherapy for each sex. **C** Percentage of no delayed vomiting during 24–120 h after chemotherapy for each sex. **D** Percentage of no delayed nausea during 24–120 h after chemotherapy for each sex. Light gray bars represent male patients and dark gray bars represent female patients

As shown in Table 2, in the acute phase, a total of 94/94 (100%) of the CQ in the male group versus 76/82 (92.7%) of the CQ in the female group remained emesis-free (*p* = 0.009); and 86/94 (91.5%) of the CQ in the male group versus 65/82 (79.3%) of the female group remained nausea-free (*p* = 0.021). In the delayed phase, results showed that 93/94 (98.9%) of the CQ in the male group versus 78/82 (95.1%) of the CQ in the female group were vomits-free (*p* = 0.186); and regarding nausea, 85/94 (90.4%) of the CQ in male group versus 65/82 (79.3%) of the CQ in the female group not experienced nausea (*p* = 0.037). Figure 1 represents the results in each group according to the CINV phase.

**Table 2:** CINV results in the acute and delayed phases according to sex

Overall results	Male (*n* = 94)		Female (*n* = 82)		*P*
	*n* (%)	Median [IQR]	*n* (%)	Median [IQR]	
**Acute phase**					
**Emesis**					
No (%)	94 (100)		76 (92.7)		0.009^**^
Yes (%)	0 (0)	-	6 (7.3)	1.5 (1–2)	
**Nausea**					
No (%)	86 (91.5)		65 (79.3)		0.021^*^
Yes (%)	8 (8.5)	3 (2–9.5)	17 (20.7)	3 (2–5)	
**Delayed phase**					
**Emesis**					
No (%)	93 (98.9)		78 (95.1)		0.186
Yes (%)	1 (1.1)	1 (1–1)	4 (4.9)	1.5 (1–5)	
**Nausea**					
No (%)	85 (90.4)		65 (79.3)		0.037^*^
Yes (%)	9 (9.6)	5 (3–9)	17 (20.7)	4 (2–5)	
**High emetic risk chemotherapy** (*n* = 53)	*n* (%)		*n* (%)		
**Acute phase**					
**Emesis**					
No (%)	53 (100)		33 (91.7)		0.063
Yes (%)	0 (0)		3 (8.3)		
**Nausea**					
No (%)	50 (94.3)		27 (75.0)		0.012^*^
Yes (%)	3 (5.7)		9 (25.0)		
**Delayed phase**					
**Emesis**					
No (%)	53 (100)		35 (97.2)		0.404
Yes (%)	0 (0)		1 (2.8)		
**Nausea**					
No (%)	49 (92.5)		26 (72.2)		0.010^**^
Yes (%)	4 (7.6)		10 (27.8)		
**Moderate emetic risk chemotherapy** (*n* = 22)	*n* (%)		*n* (%)		
**Acute phase**					
**Emesis**					
No (%)	22 (100)		16 (94.1)		0.436
Yes (%)	0 (0)		1 (5.9)		
**Nausea**					
No (%)	22 (100)		17 (100)		-
Yes (%)	0 (0)		0 (0)		
**Delayed phase**					
**Emesis**					
No (%)	21 (95.5)		16 (94.1)		1.0
Yes (%)	1 (1.1)		1 (5.9)		
**Nausea**					
No (%)	21 (95.5)		16 (94.1)		1.0
Yes (%)	1 (1.1)		1 (5.9)		
**Low emetic risk chemotherapy** (*n* = 19)	*n* (%)		*n* (%)		
**Acute phase**					
**Emesis**					
No (%)	19 (100)		27 (93.1)		0.512
Yes (%)	0 (0)		2 (6.9)		
**Nausea**					
No (%)	14 (73.7)		21 (72.4)		0.923
Yes (%)	5 (26.3)		8 (27.6)		
**Delayed phase**					
**Emesis**					
No (%)	19 (100)		27 (93.1)		0.512
Yes (%)	0 (0)		2 (6.9)		
**Nausea**					
No (%)	15 (79.0)		23 (79.3)		1.0
Yes (%)	4 (21.0)		6 (20.7)		

^*^P ≤ 0.05, ^**^P ≤ 0.01were considered statistically significant

The results of CINV according to chemotherapy emetic potential are summarized in Table 2. In patients with low and moderate emetic potential, no statistically significant differences were found between groups. Thirty-seven patients (89 CQ), with high emetic chemotherapy reported a higher incidence of nausea in females than in males, 25.0 versus 5.7% for acute and 27.8 versus 7.6% for delayed.

The proportion of patients who had non-CR was 11.7% for males and 32.9% for females (*p* = 0.001). No patient required a modification of the antiemetic regimen due to non-CR. The odds ratios (ORs) and 95% CIs of patient-related risk factors for a non-CR in the overall cohort and according to sex are displayed in Table 3. Univariate and multivariate logistic regression analysis revealed that sex and young age were significantly associated with a non-CR in the overall cohort (sex OR_univariate_, 3.70; 95%CI, 1.70–8.08; *p* = 0.001; OR_multivariate_, 4.45; 95%CI, 1.58–12.55; *p* = 0.005; young age OR_univariate_, 2.74; 95%CI, 1.22–6.17; *p* = 0.017; OR_multivariate_, 4.11; 95%CI, 1.39–12.19; *p* = 0.011), while schemes with a moderate emetic risk may contribute to reducing the risk of non-CR compared with low emetic risk (OR_univariate_, 0.15; 95%CI, 0.04–6.17; p = 0.005; OR_multivariate_, 0.11; 95%CI, 0.03–0.66; *p* = 0.014). Habitual smoking reduced the risk of non-CR but only was statistically significant in the univariate analysis. The rest of the factors were not associated with a non-CR in the overall population. In the male group, there were no factors associated with a non-CR, while in females, young age contributed to a non-CR (OR_univariate_, 4.21; 95%CI, 1.22–14.49; *p* = 0.020; OR_multivariate_, 26.95; 95%CI, 2.57–282.35; *p* = 0.006), as well as a previous pregnancy-related nausea and vomiting history but only was significant in the univariate analysis (OR_univariate_, 6.96; 95%CI, 2.23–21.74; *p* = 0.001).

**Table 3:** Patient-related risk factors associated with treatment failure in overall phase and according to sex

Overall results	Univariate Analysis		Multivariate Analysis	
Variable	OR (95% CI)	*P*	OR (95% CI)	*P*
Sex (female)	3.70 (1.70–8.08)	0.001	4.45 (1.58–12.55)	0.005
Age (< 60 years)	2.74 (1.22–6.17)	0.017	4.11 (1.39–12.19)	0.011
Motion sickness (yes)	1.84 (0.16–20.83)	0.636	0.65 (0.05–8.31)	0.744
Habitual alcohol intake (yes)	0.35 (0.10–1.23)	0.069	0.74 (0.11–4.87)	0.758
Habitual smoking (yes)	0.21 (0.05–0.92)	0.012	0.19 (0.03–1.01)	0.051
Emetic risk				
Moderate	0.15 (0.04–0.57)	0.005	0.11 (0.03–0.66)	0.014
High	0.46 (0.21–1.01)	0.054	0.29 (0.16–1.57)	0.189
Treatment line (first line)	0.84 (0.41–1.74)	0.645	0.52 (0.32–2.80)	0.929
ECOG				
1	1.42 (0.63–3.25)	0.398	1.34 (1.02–7.13)	0.046
2	1.96 (0.51–7.52)	0.327	3.28 (0.64–21.16)	0.145
**Male results**				
Age (< 60 years)	3.23 (0.88–11.88)	0.085	1.94 (0.28–13.22)	0.501
Motion sickness (yes)			-	
Habitual alcohol intake (yes)	0.23 (0.03–1.91)	0.108	1.05 (0.07–14.88)	0.971
Habitual smoking (yes)			-	
Emetic risk				
Moderate	0.10 (0.11–0.96)	0.046	0.10 (0.04–2.62)	0.168
High	0.18 (0.4–0.72)	0.016	0.13 (0.01–1.87)	0.132
Treatment line (first line)	0.71 (0.19–2.65)	0.618	0.27 (0.02–3.05)	0.290
ECOG				
1	0.62 (0.17–2.23)	0.467	0.75 (0.11–5.05)	0.769
2			-	
**Female results**				
Age (< 60 years)	4.21 (1.2–14.49)	0.020	26.95 (2.57–282.35)	0.006
History of nausea and vomiting during pregnancy (yes)	6.96 (2.23–21.74)	0.001	2.00 (0.26–15.46)	0.507
Motion sickness (yes)	1.02 (0.09–11.76)	0.988	0.37 (0.01–8.07)	0.526
Habitual alcohol intake (yes)	2.12 (0.28–15.93)	0.470	0.39 (0.01–12.43)	0.596
Habitual smoking (yes)	0.47 (0.09–2.38)	0.335	0.07 (0.004–1.16)	0.063
Emetic risk				
Moderate	0.22 (0.04–1.14)	0.071	0.08 (0.004–1.75)	0.110
High	1.04 (0.38–2.85)	0.937	0.57 (0.05–5.84)	0.636
Treatment line (first line)	1.49 (0.59–3.78)	0.394	0.87 (0.14–5.53)	0.882
ECOG				
1	2.77 (0.88–8.70)	0.081	11.49 (1.1–119.70)	0.041
2	2.93 (0.60–14.45)	0.186	21.89 (0.73–652.87)	0.075

*OR* odds ratio

Regarding the assessment of the most frequent adverse events caused by antiemetics, both sexes reported more frequently having constipation, followed by insomnia in the first 5 days after chemotherapy. Females suffered more from constipation than males (52.4 versus 37.2%, *p* = 0.043), as shown in Table 4.

**Table 4:** Antiemetic adverse effects recorded according to sex

	Male (*n* = 94)	Female (*n* = 82)	*P*
Constipation	35 (37.2)	43 (52.4)	0.043^*^
Insomnia	19 (20.2)	17 (20.7)	0.932
Headache	11 (11.7)	7 (8.5)	0.489

^*^P ≤ 0.05 was considered statistically significant

## Discussion

To our knowledge, this is the first prospective study specifically designed to assess the incidence of CINV among both sexes. We showed that females have a higher incidence of CINV, especially acute emesis and acute and delayed nausea. These differences were especially notable in the high emetic potential regimens, probably influenced by the larger sample size and because CINV symptomatology is most aggravated in this subgroup in females. Female sex and young age were shown to be clear predictors of non-CR, while habitual smoking and moderate emetic risk schemes may reduce the frequency of CINV. In males, no predictors of non-CR were identified, whereas in females, young age and pregnancy-related nausea and vomiting contributed to it. A high percentage of patients reported adverse effects, constipation being the most common one, especially in females.

It is well-known that the incidence of CINV differs based on sex, as has been proven in different studies [16, 19, 24–29]. Sekine et al. and Hilarius et al. demonstrated a higher incidence of CINV in females, both in the acute and delayed phases, as well as a relationship between the number of risk factors and the occurrence of CINV [5, 16]. Binder et al. confirmed that CINV rates were higher in young females than in males [28]. Zhao et al. evaluated the incidence of CINV in highly emetic chemotherapy, proving that females with no history of alcohol intake and a larger body surface area undergoing high doses (≥ 70 mg/m^2^) of cisplatin were more vulnerable to chemotherapy-induced nausea [29]. Uchida et al. found female sex, age < 60 years, no habitual alcohol intake, and ECOG score of 1 to be significant risk factors associated with non-CR [25]. Tsuji et al. in patients treated with highly emetic chemotherapy, revealed that sex and age, among other factors, were significant and independent factors affecting CINV in the overall phase [26]. The systematic review and meta-analysis conducted by Mosa et al. analyzed the influence of sex in 32 studies, and of those, 18 studies confirmed that female patients are at higher risk of CINV than male patients (*p* < 0.05), showing a summary odds ratio of 2.79 (95%CI 2.26–3.44) [24]. In our study, the OR in the overall cohort was even higher, 3.70 (95%CI 1.70–8.08) in the univariate analysis and 4.45 (95%CI 1.58–12.55) in the multivariate analysis, confirming the results of the meta-analysis and previous studies.

Young females are at high risk of suffering CINV, especially those who suffer from emesis and nausea during pregnancy. It may be due to nausea and vomiting, usually caused by conditioned stimuli. Patients with a prior history of this effect are at higher risk of suffering it again when they are exposed to the same stimuli [14]. In addition, aging in women seems to be a factor that decreases the incidence of CINV. Menopause has been shown to influence the severity of nausea; probably, changes in FSH and E2 due to menopause may affect the severity of nausea [30]. In our study, this hormonal status was not collected. However, according to the mean age of the female patients, it is expected that most of them were post-menopausal. It is possible that the differences between groups would be even greater if the female patients were younger, since young age was one of the main determinants of CINV.

In the latest version of the ESMO guidelines [31], the recommendations for CINV prevention due to high emetic chemotherapy based on anthracycline-cyclophosphamide (AC), were differentiated according to gender. In women treated with AC-based chemotherapy, a four-drug regimen including single doses of 5-HT_3_-RA, dexamethasone, NK_1_-RA, and olanzapine before chemotherapy is recommended to prevent acute CINV, and the addition of olanzapine to a triple antiemetic association on days 2–4 is suggested to prevent delayed nausea and vomiting. Similarly, for the prevention of acute CINV following oxaliplatin-based moderate chemotherapy, the addition of an NK_1_-RA before chemotherapy is suggested for women aged < 50 years old against a two-drug regimen (5-HT_3_-RA and DEX), recommended for the rest of patients receiving oxaliplatin [31]. These changes in treatment guidelines from international organizations such as ESMO highlight the increasing gender differences in CINV and the need for individualized approaches. In patients, especially women, with poor CINV control, different strategies can be employed, such as the addition of olanzapine, which has been shown to significantly improve CR rates as well as CINV prevention in chemotherapy-naive patients [32]. Other less-studied strategies have been using oral cannabinoids such as dronabinol and nabilone, which may have a role in controlling emesis from a neurophysiological perspective. Those drugs appear to offer a useful additional option, but further studies are still required to evaluate their efficacy and safety in the case of difficult-to-control CINV [33].

Other factors like habitual alcohol intake and habitual smoking have been described in previous studies to reduce the incidence of CINV [16, 24, 27, 34, 35]. In our study, only habitual smoking seemed to contribute to reducing CINV in the overall population, but only in the univariate analysis, like the study of Sekine et al. [16]. Another well-described factor is a history of pregnancy-related nausea and vomiting. In females, this factor has been associated with an increased risk of vomiting [35]. In our study, this factor was also found to increase the risk of CINV, although only in the univariate analysis.

Personal predisposing factors such as sex, age, habitual smoking, and history of pregnancy-related nausea and vomiting seem to be the main contributors to CINV in our study. The emetic potential of chemotherapy seems not to play such an important role, unlike what has been reported in previous studies and current antiemetic guidelines [8, 11, 13]. In fact, in our study, as the emetic potential of the chemotherapy regimen increased, the risk of CINV lowered; OR moderate emetic risk 0.15 (0.04–0.57), OR high emetic risk 0.46 (0.21–1.01). These data suggest that it is possible to predict in advance that patients are at high risk of CINV to adjust their antiemetic prophylaxis and that patients with a low emetic risk chemotherapy are not well protected against CINV. In addition, it was observed that approximately half of the patients who experienced CINV did so in more than one cycle, which further highlights the importance of personal predisposing factors in CINV. It is therefore important that the antiemetic regimen considers predisposing risk factors beyond the emetic risk of the chemotherapy scheme [6, 7, 16, 24, 28]. This might help to select high-risk patients for whom more potent antiemetic regimens should be prescribed and the selection of low-risk patients for whom expensive antiemetics are unlikely to be helpful and can cause them some adverse effects [35]. Different scores have been developed to accurately identify patients at high risk for CINV [36–38].

Our study recorded the incidence of the adverse effects most described in the prescribing information of the antiemetics used for chemotherapy prophylaxis. Constipation was the most reported adverse effect in both groups, being significantly the incidence being higher in females (52.4%) than in males (37.2%). Other common adverse effects reported by patients were tiredness, generalized pain, malaise, and diarrhea. Nevertheless, it remains uncertain whether these adverse effects stem solely from the administration of antiemetic agents or if they are influenced by the concurrent effects of chemotherapy. It is plausible that the interplay between chemotherapy and the patient’s psychological state of anxiety contributes to the exacerbation of adverse effects attributed to antiemetic drugs.

Our research provides valuable information on how to manage CINV according to sex, allowing early identification of patients at higher risk and individualizing antiemetic prophylaxis according to patient characteristics to reduce the incidence of antiemetic adverse effects. Nevertheless, several limitations must be acknowledged when interpreting these results. First, the data came from a single center. Hence, the findings might not apply to other settings owing to the center effect. Second, the study was non-randomized. Patients were divided into groups according to sex. The groups were balanced, although the male group was slightly larger than the female group. Third, numerous potential risk factors have been identified in the literature, some of which have been difficult to quantify for this study. Some of these factors may play a role in the observed outcomes. Biomarkers for CINV and the genetic and molecular mechanisms were not explored. It will be worthwhile to assess those factors in the future. Fourth, our study included a small percentage of patients who had already received other lines of treatment and for whom it was not registered whether they had previously suffered CINV. A history of experiencing CINV in prior treatment lines may predispose individuals to CINV in the current course of treatment. Nevertheless, that group only represented 4.3% of males and 19.6% of females. Additional multicentric studies in larger populations are needed to confirm our results and to develop more individualized regimens based on each patient’s risk according to sex. Based on our results, it would be interesting to evaluate in future studies whether men might require less potent antiemetic prophylaxis despite taking highly emetic chemotherapy. It would also be beneficial to focus on pharmacogenetics studies to investigate the role of genetic variants in selecting appropriate genetic risk factors for tailored emetic prophylaxis in clinical practice.

## Conclusion

In conclusion, the current study suggests that males and females do not experience CINV in the same way. Females experienced more from this unpleasant effect with the unpleasant consequences that it entails. In males, there were no clear predisposing factors, while in females at a young age, a previous history of nausea and vomiting during pregnancy were clear determinants for CINV. The data obtained suggest a revision of current antiemetic prophylaxis and personalization of these treatments according to the sex and age of patients.

## Data Availability

No datasets were generated or analysed during the current study.
